# Analyses of human immune responses to *Francisella tularensis* identify correlates of protection

**DOI:** 10.3389/fimmu.2023.1238391

**Published:** 2023-09-15

**Authors:** Helena Lindgren, Kjell Eneslätt, Igor Golovliov, Carl Gelhaus, Anders Sjöstedt

**Affiliations:** ^1^ Department of Clinical Microbiology, Umeå University, Umeå, Sweden; ^2^ Appili Therapeutics, Halifax, NS, Canada

**Keywords:** *F. tularensis*, vaccination, immune response, memory cells, human correlates of protection

## Abstract

*Francisella tularensis* is the etiological agent of the potentially severe infection tularemia. An existing *F: tularensis* vaccine, the live vaccine strain (LVS), has been used to protect at-risk personnel, but it is not licensed in any country and it has limited efficacy. Therefore, there is a need of a new, efficacious vaccine. The aim of the study was to perform a detailed analysis of the characteristics of the human immune response to *F. tularensis*, since this will generate crucial knowledge required to develop new vaccine candidates. Nine individuals were administered the LVS vaccine and peripheral blood mononuclear cells (PBMC) were collected before and at four time points up to one year after vaccination. The properties of the PBMC were characterized by flow cytometry analysis of surface markers and intracellular cytokine staining. In addition, the cytokine content of supernatants from *F. tularensis*-infected PBMC cultures was determined and the protective properties of the supernatants investigated by adding them to cultures with infected monocyte-derived macrophages (MDM). Unlike before vaccination, PBMC collected at all four time points after vaccination demonstrated *F. tularensis*-specific cell proliferation, cytokine secretion and cytokine-expressing memory cells. A majority of 17 cytokines were secreted at higher levels by PBMC collected at all time points after vaccination than before vaccination. A discriminative analysis based on IFN-γ and IL-13 secretion correctly classified samples obtained before and after vaccination. Increased expression of IFN-γ, IL-2, and MIP-1β were observed at all time points after vaccination vs. before vaccination and the most significant changes occurred among the CD4 transient memory, CD8 effector memory, and CD8 transient memory T-cell populations. Growth restriction of the highly virulent *F. tularensis* strain SCHU S4 in MDM was conferred by supernatants and protection correlated to levels of IFN-γ, IL-2, TNF, and IL-17. The findings demonstrate that *F. tularensis* vaccination induces long-term T-cell reactivity, including T_EM_ and T_TM_ cell populations. Individual cytokine levels correlated with the degree of protection conferred by the supernatants. Identification of such memory T cells and effector mechanisms provide an improved understanding of the protective mechanisms against *F. tularensis*. mechanisms against *F. tularensis*.

## Introduction

Tularemia is a disease caused by *Francisella tularensis* that affects many animal species as well as humans. The most severe form of tularemia in humans, with high mortality if untreated, is due to inhalation of *F. tularensis* subspecies *tularensis* (type A) (1). The vaccine against *F. tularensis*, a live vaccine of the less virulent subspecies *holarctica* (LVS), offers limited protection against this form of tularemia (2). Moreover, since the knowledge regarding the attenuation of LVS is incomplete, the vaccine is not licensed in any country. Tularemia is widespread over the Northern Hemisphere with local, unpredictable outbreaks, although rather uncommon in many countries (1). The highest total number of cases has been reported in Sweden, Finland, Turkey, Hungary, and Czech Republic. In these countries, as well as in other European countries, there are very marked annual and seasonal variations (3, 4). Although not a threat to the public health in most countries, there are endemic areas in, for example, Sweden and Finland with persistent high incidences, and in these areas, an efficacious vaccine would be of much value. In addition, there are at-risk groups that would benefit from vaccination, for example, clinical laboratory personnel and individuals regularly working outdoors, such as farmers.

*F. tularensis* is classified as a Category A Select Agent, due to its ease of spread by aerosol, extremely low infectious dose, and potential to cause severe morbidity and mortality, therefore considered to have the potential to pose a severe threat to public health and safety (1). The high virulence of *F. tularensis* relies on its ability to proliferate in many different cell types, including macrophages (5). As a consequence, a Th1-dependent cellular immune response is evoked to protect against the bacterium (6, 7). Detailed analysis of the characteristics of the immune response to *F. tularensis* has been performed by *in vivo* and *in vitro* studies, mostly using mice and rats, but also *ex vivo* studies using human cells (8–14). The efficacy of new vaccine candidates has been tested in mice, rats, and non-human primates, but human clinical trials are unlikely due to the low incidence and unpredictability of tularemia (7, 15). Thus, our knowledge regarding the human immune response to *F. tularensis* is based mostly on *ex vivo* studies of peripheral blood mononuclear cells (PBMCs) derived from immune individuals.

Studies of vaccine-mediated immune responses have demonstrated that there is an initial phase of rapid proliferation and expansion of antigen-specific T-cell clones and a majority of circulating T cells are antigen-specific. Subsequently, the responding cells contract and form a much smaller memory immune population (16). Experimental models have also demonstrated that the specificities of the T-cell responses are complex and composed of distinct epitope specificities with hierarchies of dominant, subdominant, and cryptic responses (17). The immunospecific T cells are characterized by their expression of surface receptors and markers, which are believed to identify the T-cell differentiation stage. However, the division of T-cell subpopulations into naïve, memory effector, and memory immunity T cells may not be unequivocal, since there is evidence that the expression of surface receptors and markers and their correlation to effector properties may vary between different infections and vaccinations (18, 19). This indicates that studies of specific infection models are necessary to identify the pathogen-specific T cells that constitute the long-term memory as well as those that effectuate the anti-microbial responses. By combining data on the specificities of responding T cells with characterization of their phenotypes, it will be possible to delineate the responsible subpopulations and to determine their reactivity.

In addition to vaccine-specific variations in the memory immune responses, there is always heterogeneity due to individual variation and some individuals may not mount an effective immune response to certain vaccines (18). Such differences can be utilized to understand what best protects against a given pathogen. The present study analyzed development of memory T cells and cytokine responses before and after *F. tularensis* vaccination. Furthermore, it was assessed how such variation affected the protective efficacy by analyzing the ability of supernatants collected from recall-stimulated PBMC to provide control of *F. tularensis* in monocyte cultures. Thereby, it was possible to identify cytokines and memory cells of importance for protection against highly virulent strains of *F. tularensis.*

## Materials and methods

### Vaccination

All individuals were vaccinated the same day with a variant of LVS designated NDBR 101, lot no. 11 (National Drug Company, Philadelphia, PA). The lyophilized material was dissolved in 2.0 ml of sterile H_2_O to a concentration of 2.4 × 10^9^ CFU/ml and 20 µl was inoculated by scarification in the skin of the upper arm. Ethical approvals for the study were received from the Swedish Ethical Review Authority, 2019-01567 and 2020-01860.

### Preparation of PBMCs from blood

Venous blood was drawn from healthy individuals, five women and four men (22–53 years old at time of vaccination, mean age 36.3 ± 12.2 years) before vaccination and 2, 4, 12, 52, and 104 weeks after vaccination. The blood, approximately 100 ml, was collected in CPT-tubes (BD Biosciences NJ USA) and PBMCs were prepared according to the manufacturer’s protocol. Purified PBMC was suspended in human serum (HS) (Innovative research, MI, USA) containing 10% DMSO (Sigma Aldrich, MO, USA) and aliquoted into cryovials, which was placed in a Cryo 1°C Freezing Container (NALGENE, NY, USA) at −80°C overnight before transferred into liquid nitrogen.

### Recall stimulation PBMC

Cryovials with PBMC were thawed in a 37°C water bath and transferred to 20 ml of RPMI medium 1640 + GlutaMAX (RPMI), (Gibco, MA USA). The PBMC was collected by centrifugation at 200 × *g* for 10 min, washed with 40 ml of RPMI, and suspended in 1 ml of RPMI + 10% HS + 10 µg/ml Gentamicin (complete RPMI). After resting at 37°C in 5% CO_2_ for 2 h, the PBMCs were counted and diluted in complete RPMI. For FACS analysis, 8 × 10^5^ cells were seeded per well in a round-bottom 96-well plate (Sarstedt, Nümbrecht, Germany). For the Lymphocyte proliferation assay (LPA), 2 × 10^5^ cells were seeded per well. To some wells, *Ft* antigen (2.5 µg/ml), prepared from *F. tularensis* LVS as described previously (20) or Concavalin A (ConA) (2.5 µg/ml), was added (stimulated cells) whereas other wells contained complete RPMI only (resting cells). After 3 days of incubation, 300,000–500,000 cells per sample were collected and analyzed by FACS. From separate wells, supernatants were transferred to a 96-well plate and stored at −80°C until analyzed for cytokine content by multiplex cytokine analysis. For LPA, tritium-thymidine (0.003 mCi/ml) (Perkin Elmer MA USA) was added to the cells, and after 6 h, incorporated thymidine was measured using a 1450 microbeta liquid scintillation & luminescence counter (Trilux Chelmsford UK). All time points from two to three individuals were included in each experiment.

### Flow cytometry analysis of surface markers and intracellular cytokine staining

Cells were collected after 72 h of recall stimulation and treated with 5 µg/ml of Brefeldin A and 5 µg/ml of Monensin for 4 h. Then, cells were centrifuged for 3 min at 500 × *g* and supernatants were removed. Cells were stained with Aqua Viability Dye (Molecular Probes/Invitrogen) for 20 min in RT and thereafter labeled with conjugated monoclonal antibodies (mAbs) against cell surface markers for 30 min at 4°C. After wash and treatment with perm/wash buffer (BD Biosciences) for 20 min at 4°C, the cells were stained for intracellular cytokines for 30 min at 4°C. The following mAb conjugates were used (BD Biosciences): CD3-APCH7 (clone SK7), CD4-FITC (clone RPA-T4), CD8-PerCPCy5.5 (clone SK1), CD45RO-APC (clone UCHL-1), CCR7-PECF594 (clone 2-L1-A), CD28-PE (clone CD28.2), CD95-BUV395 (clone DX2), IFNγ-PeCY7 (clone B27), MIP-1β-AF700 (clone D21-1351), IL2-BV711 (clone 5344.111), and TNF-BV421 (clone MAb11). CD14-V500 (clone M5E2) and CD19-V500 (clone H1B19) were included in the dump channel. PBMCs were acquired using a ZE5 flow cytometer (Bio-Rad) with Everest software (Bio-Rad). Results were analyzed using FlowJo software (BD Biosciences).

### Multiplex cytokine analysis

Supernatants, 50 μl/well, were collected from cultures after 72 h of incubation. The supernatants were stored at −80°C until analyzed using a 17-plex kit (Bio-Rad Laboratories Inc., Hercules, CA, USA, M5000031YV) according to the manufacturer’s instructions using a Bio-Plex 200 system (Bio-Rad Laboratories Inc., Hercules, CA, USA).

### Isolation of monocytes and generation of monocyte-derived macrophages

PBMCs, 15 × 10^6^ cells, prepared from buffy coat, were seeded in a 9-cm petri dish in 15 ml of complete RPMI. After 2 h, medium containing non-adherent cells was removed and the adherent cells were washed with 20 ml of 37°C RPMI. The adherent cells were detached by scraping and thereafter transferred to a 50-ml tube, which was centrifuged at 200 × *g* for 10 min. The cells were suspended in complete RPMI, viable cells were determined using trypan blue exclusion in a TC20 cell counter (Bio-Rad Laboratories Inc, Hercules, CA, USA), and 1 × 10^5^ cells were seeded per well in a flat-bottom 96-well culture plate. After overnight incubation at 37°C in 5% of CO_2_, cells were washed and thereafter complete RPMI with 40 ng/ml of GM-CSF was added. After 48 h incubation at 37°C in 5% CO_2_, cells were washed and complete RPMI with 40 ng/ml of GM-CSF was added. Complete RPMI (100 µl) was added to each well after an additional 48 h of incubation.

### Infection of MDMs

SCHU S4, grown overnight on Gc-agar plates, were resuspended in complete RPMI and added to the MDM monolayer at an MOI of 50. After 1 h, cells were washed and complete RPMI with 10 µg/ml of gentamicin was added for 30 min. To some wells, 20-fold diluted supernatant, collected from *Ft*-stimulated PBMCs from respective donor, was added to separate cultures. Complete RPMI was used as diluent. The number of intracellular bacteria was determined after 24 h lysis of the monolayers with 0.1% deoxycholate and spreading of 10-fold serial dilutions of the lysate in PBS on agar plates. Colonies were counted after 3 days of incubation of the plates at 37°C in 5% CO_2_.

### Data analysis and statistical methods

Two-tailed Student’s *t*-test was used to identify significant differences (*p* < 0.05) between data sets. To analyze correlations between data sets, Spearman’s rank correlation test was used. Cytokine data were used to derive a classifier that enables prediction of vaccination status, i.e., to predict if PBMCs were derived from individuals before vaccination with LVS or derived at 2, 4, 12, or 52 weeks after vaccination. Linear discriminant analysis (LDA), assuming homoscedasticity and no prior, was used to build the classifiers and cross-validation was used to predict the posterior probabilities (21). The LDA analyses were performed using the settings discriminant analysis and stepwise method (Wilks´ Lambda) with the criteria of F to enter 3.84 and F to remove 2.71 in the program SPSS version 28. SPSS was also used to perform two-tailed Student’s *t*-test and Spearman’s rank correlation test.

## Results

### Proliferative responses of PBMCs

**Figure 1:**
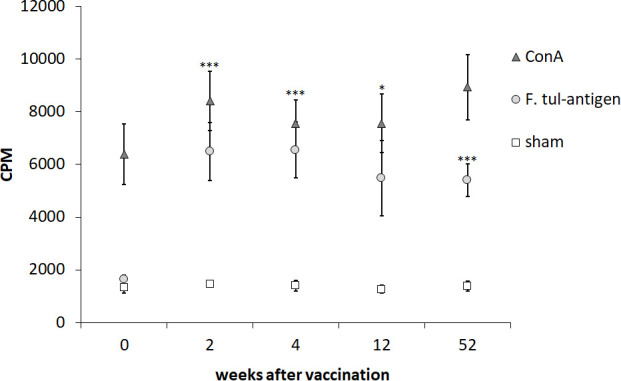
Proliferative responses of PBMCs after recall stimulation with *Ft* antigen. PBMCs collected from nine individuals before vaccination (0) and 2, 4, 12, and 52 weeks after vaccination were cultured in the presence or absence of *Ft* antigen for 3 days. Six hours after addition of tritium-thymidine, incorporation was measured. The proliferative responses of the sham, ConA-stimulated, or *Ft*-stimulated cells are expressed as CPM for each time point. The mean ± SEM of triplicate samples from nine individuals are shown for each time point. Asterisks indicate significant differences of the *Ft*-stimulated groups compared to *Ft*-stimulated cells obtained before vaccination, denoted week 0 (*P < 0.05; ***P < 0.001). There were no significant differences between the time points after vaccination for each of the stimuli.

PBMCs isolated from nine individuals before vaccination with LVS and 2, 4, 12, and 52 weeks after vaccination were stimulated with *Ft* antigen, ConA, or sham for 3 days. As expected, PBMCs responded to ConA with robust proliferation, whereas non-stimulated PBMCs showed minor proliferation (**Figure 1**). The average *Ft*-specific proliferative responses of PBMCs isolated from the nine individuals after vaccination, regardless of time point, were significantly higher than that of PBMCs isolated prior to vaccination (*p* < 0.05–*p* < 0.001, **Figure 1**) The magnitude of the *Ft*-specific proliferative responses varied among individuals (**Figure S1**).

In summary, the PBMCs showed robust proliferation to ConA, whereas only PBMCs collected after vaccination proliferated in response to the *Ft* antigen.

### Detection of intracellular cytokines

**Figure 2:**
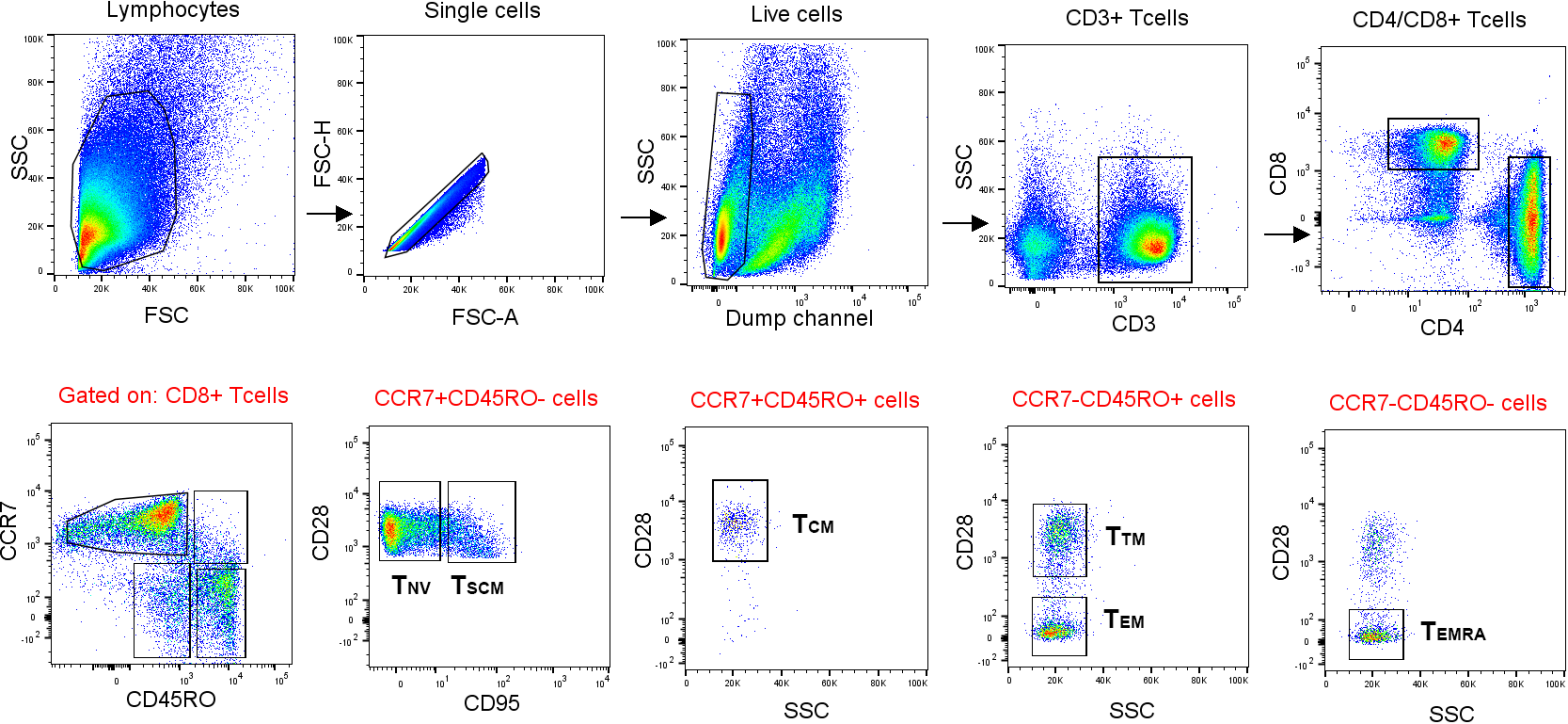
Gating strategy used for FACS analysis of memory populations. Lymphocytes were gated based on morphology detected with forward and side scatter, FSC and SSC. After gating for singlets, a gate for CD14^−^ and CD19^−^ live cells was created. CD4^+^ and CD8^+^ T cells were gated from CD3^+^ T cells. The CD45RO^-^CCR7^+^ population were gated into naïve (T_NV_) and stem cell-like memory T cells (T_SCM_) by the expression of CD95. Memory populations [central memory (T_CM_), transitional memory (T_TM_), effector memory (T_EM_), and effector memory RA^+^ (T_EMRA_) cells] were further gated according to the positive or negative expression of CD45RO, CCR7, and CD28. Finally, IFN-γ-, TNF-, MIP-1β-, and IL-2-expressing cells were gated from the naïve and memory populations, respectively.

PBMCs stimulated with the *Ft* antigen for 3 days, or sham-stimulated PBMCs, were analyzed by FACS for intracellular expression of IFN-γ, IL-2, MIP-1β, or TNF. In previous studies, these cytokines have been identified to discriminate responses of immune vs. naïve individuals (12, 22). The cells were also stained for cell surface markers, which enabled identification of CD4 and CD8 lymphocytes and of various memory populations thereof (**Figures 2**, **S2**, **S3** illustrate the gating strategies).

**Figure 3:**
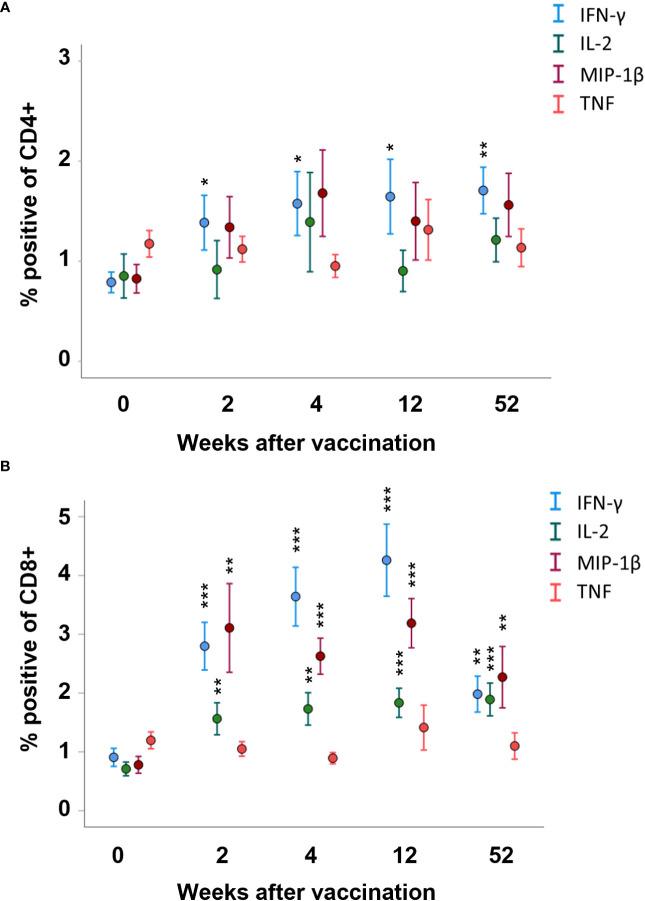
FACS analysis of intracellular cytokine expression of the CD4 and CD8 memory populations. PBMCs collected from nine individuals before vaccination (0) and 2, 4, 12, and 52 weeks after vaccination were recall-stimulated with *Ft* antigen for 3 days. The cells were analyzed to identify **(A)** CD4 and **(B)** CD8 memory populations for expression of IFN-γ, IL-2, MIP-1β, or TNF. The mean ± SEM of triplicate samples from nine individuals are shown for each time point and cytokine. Asterisks indicate significant differences of intracellular cytokine expression compared to time point 0. (*P < 0.05; **P < 0.01; ***P < 0.001).

The number of CD4 T cells expressing IFN-γ was higher among PBMCs collected 2, 4, 12, and 52 weeks after vaccination, compared to PBMCs collected prior to vaccination (*p* < 0.05–*p* < 0.01, **Figure 3A**). For the other cytokines, there were no significant differences among the CD4 T cells at any of the time points (**Figure 3A**). The number of CD8 T cells expressing IFN-γ, IL-2, or MIP-1β was higher among PBMCs collected 2, 4, 12, and 52 weeks after vaccination, compared to PBMCs collected prior to vaccination (*p* < 0.01 or *p* < 0.001, **Figure 3B**). The number of CD4 or CD8 T cells expressing TNF was unchanged before and after vaccination (**Figures 3A, B**). When comparing various time points after vaccination, the sole difference identified was a lower number of IFN-γ-expressing CD8 T cells at the 52-week time point compared to the 4- and 12-week time points (*p* < 0.01, **Figure 3B**).

**Figure 4:**
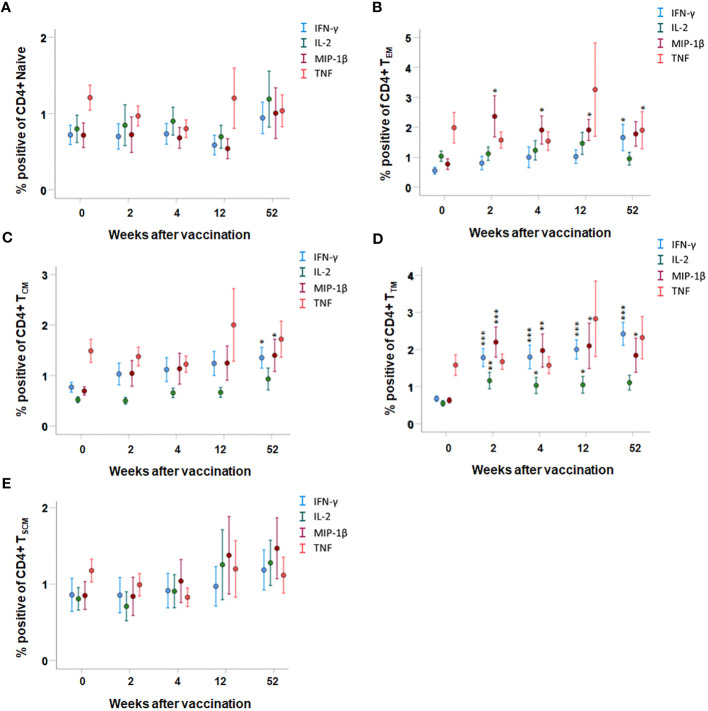
FACS analysis of intracellular cytokine expression of CD4 memory subpopulations. PBMCs collected from nine individuals before vaccination (0) and 2, 4, 12, and 52 weeks after vaccination were recall stimulated with *Ft* antigen for 3 days. The cells were stained to detect the following CD4 memory populations: **(A)** T_Naive_, **(B)** T_EM_, **(C)** T_CM_, **(D)** T_TM_, and **(E)** T_SCM_ and their expression of IFN-γ, IL-2, MIP-1β, or TNF. The mean ± SEM of triplicate samples from nine individuals are shown for each time point and cytokine. Asterisks indicate significant differences of intracellular cytokine expression compared to time point 0 (*P < 0.05; **P < 0.01; ***P < 0.001). The T_EMRA_ subpopulation was not detected in the CD4^+^ population.

Cytokine expression by various T cell memory subpopulations was also analyzed. The most significantly increased expression of cytokines among CD4 T cells was identified in the transient memory (T_TM_) population; IFN-γ-, IL-2-, and MIP-1β-expressing cells were increased at all time points after vaccination compared to prior to vaccination (*p* < 0.05–*p* < 0.001, **Figure 4D**). In the CD4 T-cell effector memory (T_EM_) population, cells expressing MIP-1β were increased at all time points after vaccination and cells expressing IFN-γ were increased at the 52-week time point after vaccination (*p* < 0.05, **Figure 4B**). The number of CD4 central memory (T_CM_) cells expressing IFN-γ or MIP-1β was higher in PBMCs collected 52 weeks after vaccination, but not at the other time points, compared to PBMCs collected prior to vaccination (*p* < 0.05, **Figure 4A**). The number of TNF-positive CD4 T cells was similar before and after vaccination, regardless of memory population (**Figures 4A–E**). Among the naïve and T_SCM_ CD4 T cells, the number of cytokine-expressing cells was similar before and after vaccination (*p* > 0.05, **Figures 4A, E**).

**Figure 5:**
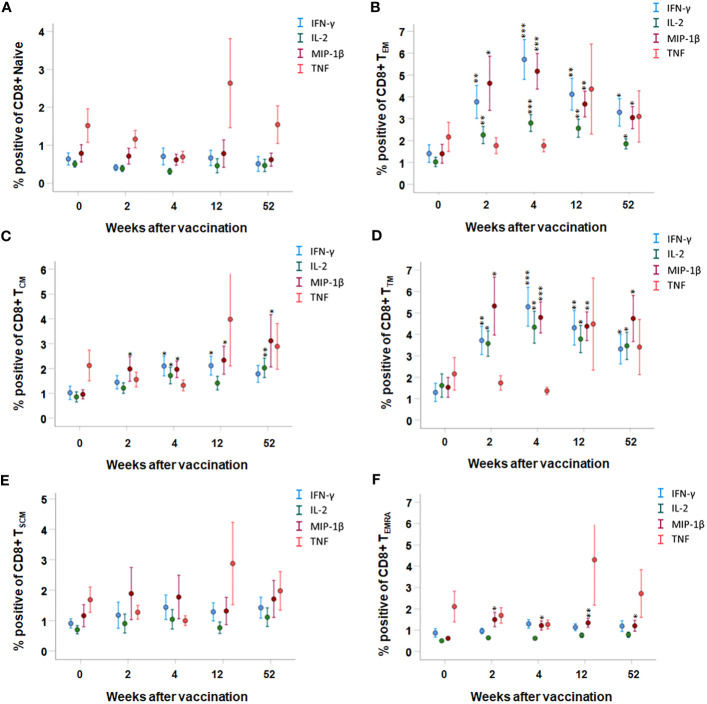
FACS analysis of the intracellular cytokine expression of CD8 memory subpopulations. PBMCs collected from nine individuals before vaccination (0) and 2, 4, 12, and 52 weeks after vaccination were recall-stimulated with *Ft* antigen for 3 days. The cells were stained to detect the following CD8 memory populations: **(A)** T_Naive_, **(B)** T_EM_, **(C)** T_CM_, **(D)** T_TM_, and **(E)** T_SCM_ and **(F)** T_TEMRA_ for their expression of IFN-γ, IL-2, MIP-1β, or TNF. The mean ± SEM of triplicate samples from nine individuals are shown for each time point and cytokine. Asterisks indicate significant differences of intracellular cytokine expression compared to time point 0. (*P < 0.05; **P < 0.01; ***P < 0.001).

In the CD8 T-cell populations, the most significant expression of cytokines was identified in the T_TM_ and T_EM_ population; IFN-γ-, IL-2-, and MIP-1β-expressing cells were increased at all time points after vaccination compared to prior to vaccination (*p* < 0.05–*p* < 0.001, **Figures 5B, D**). In addition, the number of MIP-1β expressing cells was increased at all time points after vaccination in the T_CM_ and T_EMRA_ population, and IFN-γ- and IL-2-expressing cells were increased at various time points after vaccination in the T_CM_ population (*p* < 0.05–*p* < 0.01, **Figures 5C, F**). The number of TNF-positive CD8 T cells was similar before and after vaccination, regardless of memory population (**Figures 5A–F**). Among the naïve and T_SCM_ CD8 T cells, the number of cytokine-expressing cells was similar before and after vaccination (*p* > 0.05, **Figures 5A, E**).

To verify the longevity of the memory cell populations, PBMCs from six individuals were collected 2 years after vaccination and cytokine expression by the T-cell subpopulations was analyzed. As observed at the other four time points after vaccination, CD4 T_TM_, CD8 T_TM_, and CD8 T_EM_ cell populations showed increased expression of IFN-γ, IL-2, and MIP-1β (*p* < 0.05–*p* < 0.001, **Figures S4**, **S5**).

In summary, the CD4 T_TM_, CD8 T_TM_, and CD8 T_EM_ populations showed prominently increased expression of IFN-γ, IL-2, and MIP-1β in response to vaccination for a period of at least 2 years. Increased TNF expression was not detected in any of the memory populations.

### Analysis of multifunctional cells

**Figure 6:**
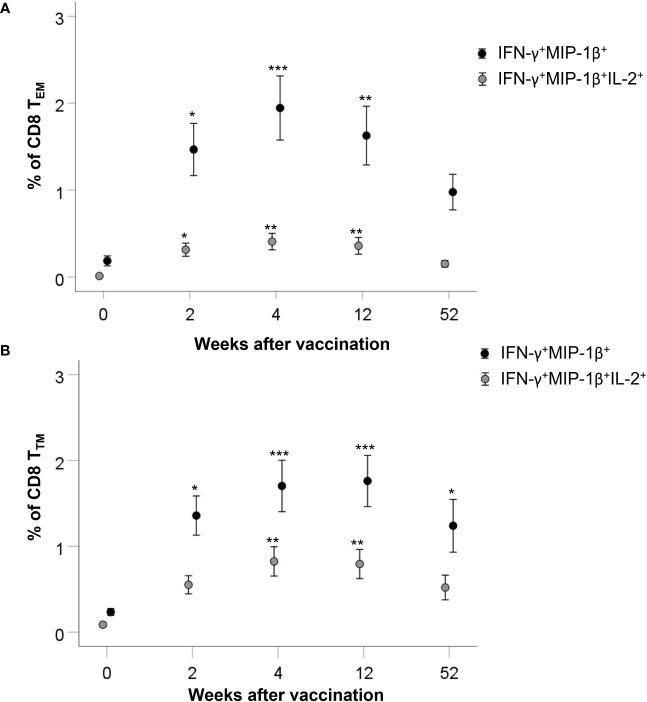
Multifunctional memory cell populations. Data were subjected to Boolean gating in order to detect multifunctional memory cell populations. The mean ± SEM of triplicate samples from nine individuals are shown for each time point and cytokine combination. Asterisks indicate significant differences of number of cells positive for intracellular cytokine expression compared to time point 0. (*P < 0.05; **P < 0.01; ***P < 0.001).

The FACS data were subjected to further analysis for the presence of multifunctional cells, i.e., cells simultaneously expressing more than one of the investigated cytokines IL-2, IFN-γ, MIP-1β, or TNF. Since their individual expression was significantly increased in the CD8 T_TM_ and CD8 T_EM_ populations, these were the focus of the analysis. Samples collected before vaccination and 2, 4, 12, and 52 weeks after vaccination were analyzed. There were significant differences for several of the groups with the highest significances (*p* ≤ 0.001) among cells expressing IFN-γ and MIP-1β, or IFN-γ, MIP-1β, and IL-2 (**Table S1**). These groups were subjected to a *post-hoc* analysis using the Bonferroni *post-hoc* test to identify differences between each of the time points (**Table S2**). The numbers of CD8 T_TM_ and CD8 T_EM_ cells expressing IFN-γ and MIP-1β were increased at all time points after vaccination compared to before vaccination (*p* < 0.05–*p* < 0.001, **Figures 6A, B**). CD8 T_EM_ cells expressing IFN-γ, MIP-1β, and IL-2 were increased up to 12 weeks (*p* < 0.05–0.001, **Figure 6A**). CD8 T_TM_ cells expressing IFN-γ, MIP-1β, and IL-2 were increased at some of the time points after vaccination (*p* < 0.05–*p* < 0.001, **Figure 6B**). At the 2-year time point, CD8 T_EM_ cells expressing IFN-γ and MIP-1β, IL-2 and MIP-1β, or IL-2, IFN-γ, and IL-2 were present in higher numbers than in samples collected before vaccination (*p* < 0.001, **Figure S6**).

In summary, CD8 T_TM_ and CD8 T_EM_ cells co-expressing various combinations of cytokines were increased post vaccination. CD8 T_TM_ and CD8 T_EM_ cells expressing IFN-γ and MIP-1B showed the most consistent increases of cytokine expression. Multifunctional CD8 T_EM_ cells were detected up to 2 years after vaccination.

### Detection of secreted cytokines and chemokines by LUMINEX

Supernatants from the *Ft* antigen-stimulated PBMCs collected prior to and after vaccination were analyzed for 17 cytokines, IL-1β, IL-2, IL-4, IL-5, IL-6, IL-7, IL-8, IL-10, IL-12p70, IL-13, IL-17, G-CSF, GM-CSF, IFN-γ, MCP-1, MIP-1β, and TNF. As aforementioned, the T_EM_ and T_TM_ cell populations demonstrated high intracellular expression of IFN-γ, IL-2, and MIP-1β, and levels of these cytokines were also high in supernatants from cultures with PBMCs collected at any of the four time points after vaccination (*p* < 0.01 or < 0.001, **Table 1**). In addition, TNF was secreted at high levels in the same cultures (*p* < 0.01). All other cytokines measured, except for IL-10, were secreted at higher levels from PBMCs collected at 52 weeks after vaccination compared to PBMCs collected before vaccination (*p* < 0.05 or *p* < 0.01, **Table 1**). Most of these cytokines were also secreted at higher levels in cultures with PBMCs collected 2, 4, and 12 weeks after vaccination (*p* < 0.05 or *p* < 0.01, **Table 1**).

**Table 1:** Cytokine levels in supernatants collected from recall-stimulated PBMCs.

Cytokine	Weeks after vaccination				
	Before	2	4	12	52
IL-1β	2.63 ± 0.11^1^	2.97 ± 0.12*^2^	3.03 ± 0.09**	3.23 ± 0.05***	3.12 ± 0.10**
IL-2	1.92 ± 0.06	2.21 ± 0.05**	2.17 ± 0.06**	2.27 ± 0.04***	2.22 ± 0.07**
IL-4	1.44 ± 0.06	1.71 ± 0.05**	1.74 ± 0.05**	1.82 ± 0.03***	1.79 ± 0.05***
IL-5	2.54 ± 0.05	2.71 ± 0.05*	2.73 ± 0.04**	2.80 ± 0.03***	2.82 ± 0.03***
IL-6	4.19 ± 0.06	4.39 ± 0.08	4.43 ± 0.08	4.49 ± 0.06**	4.61 ± 0.03***
IL-7	0.62 ± 0.18	1.60 ± 0.06***	1.62 ± 0.14***	1.46 ± 0.21**	1.56 ± 0.14***
IL-8	6.39 ± 0.29	7.19 ± 0.29	7.05 ± 0.29	6.55 ± 0.26	7.60 ± 0.26**
IL-10	1.42 ± 0.11	1.56 ± 0.06	1.63 ± 0.07	1.68 ± 0.04*	1.62 ± 0.04
Il-12p70	1.24 ± 0.19	1.77 ± 0.06*	1.80 ± 0.07*	1.93 ± 0.06**	1.79 ± 0.11*
IL-13	0.31 ± 0.10	1.25 ± 0.08***	1.24 ± 0.13***	1.36 ± 0.09***	1.23 ± 0.10***
IL-17	1.93 ± 0.08	2.21 ± 0.08*	2.24 ± 0.07**	2.37 ± 0.04***	2.31 ± 0.06**
G-CSF	2.78 ± 0.08	2.89 ± 0.06	2.99 ± 0.05*	2.95 ± 0.07	2.98 ± 0.06
GM-CSF	1.83 ± 0.12	1.95 ± 0.10	2.09 ± 0.08	2.12 ± 0.08*	2.22 ± 0.09*
IFN-γ	2.41 ± 0.09	3.75 ± 0.10***	3.80 ± 0.19***	3.98 ± 0.18***	3.82 ± 0.19***
MCP-1	4.10 ± 0.07	4.38 ± 0.03**	4.30 ± 0.06*	4.29 ± 0.05*	4.49 ± 0.06***
MIP-1β	3.80 ± 0.04	3.98 ± 0.05**	3.95 ± 0.06	3.99 ± 0.05**	4.09 ± 0.05***
TNF	3.73 ± 0.07	4.05 ± 0.06**	3.93 ± 0.07	4.03 ± 0.06**	4.05 ± 0.09**

^1^Cytokine content was determined in supernatants obtained from Ft-stimulated PBMC cultures. Average pg/ml (log_10_) ± SEM were determined from duplicate samples of seven individuals.

^2^Two-sided t-test of differences compared to cytokine levels in cultures with Ft-stimulated PBMCs collected before vaccination. *p < 0.05, **p < 0.01, and ***p < 0.001.

### Determination of vaccination status based on cytokine patterns

**Figure 7:**
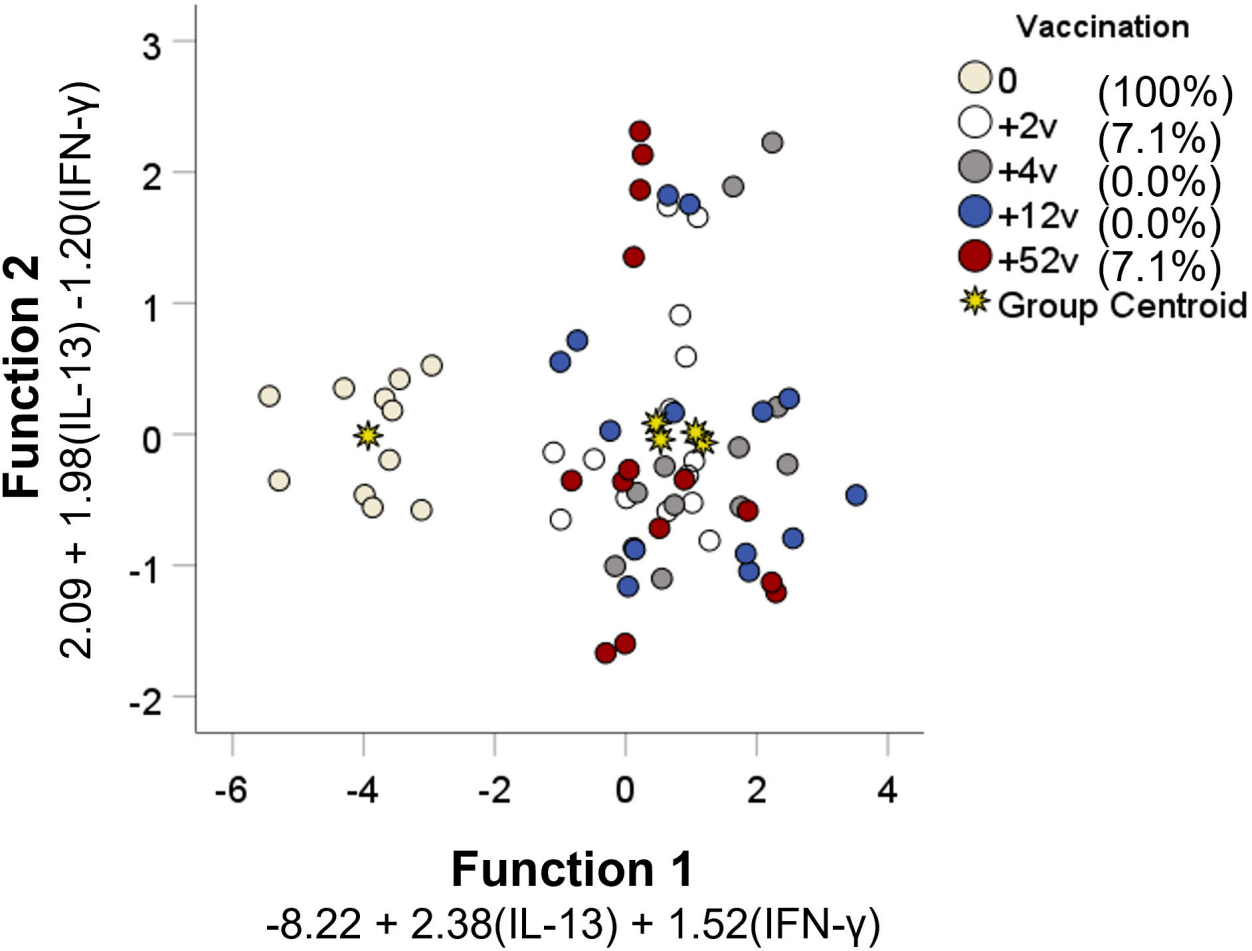
Discriminant analysis of individuals with regard to vaccination status. Seventeen cytokines measured in supernatants from recall-stimulated cell cultures were included in a stepwise discriminant function analysis to identify predictors of vaccination status. Functions 1 and 2 are depicted on the *x*- and *y*-axis, respectively. Each data point corresponds to each individual replicate of each group and the asterisks represent the group centroid. The percentage values of group classification correctness are presented in the brackets.

Linear discriminant analysis was used to determine whether individual cytokines, or sets of cytokines, could differentiate between each of the five groups: PBMCs collected before vaccination, or collected 2, 4, 12, or 52 weeks after vaccination. Two canonical discriminant functions, based on IL-13 and IFN-γ, were used in the analysis (Wilks´ Lambda 0.222, *p* < 0.001) and 99.9% of the variance was explained by function 1 (F1). The standardized canonical discriminant function coefficients for IFN-γ and IL-13 were 0.804 and 0.787 in F1, respectively, indicating that the two variables contributed similarly to the model. To further illustrate the discriminative ability of the model, the data were plotted by discriminant loading using functions 1 and 2 (**Figure 7**). The results demonstrate that non-vaccinated individuals were correctly classified in 100% of the cases and therefore visualized as a distinct group in the plot. Importantly, none of the vaccinated individuals was classified as non-vaccinated (**Table 2**). However, the resolution among the post-vaccination groups was poor (**Table 2** and **Figure 7**). As an example, the profiles of 64.3% of individuals sampled at 52 weeks overlapped with the profiles of individuals sampled at 2 weeks and there was no distinction between individuals sampled at 4 or 12 weeks (**Table 2**).

**Table 2:** Prediction of individuals according to vaccination using LDA^1^.

Predicted	Time after vaccination				
	Before	2 weeks	4 weeks	12 weeks	52 weeks
Before	100	0	0	0	0
2 weeks	0	7.1	0	57.1	35.7
4 weeks	0	0	0	50	50
12 weeks	0	42.9	42.9	0	14.3
52 weeks	0	64.3	14.3	14.3	7.1

^1^Prediction was performed based on the following: function 1: −8,223 + 2.382 (IL-13) + 1.52 (IFN-γ); function 2: 2.093 + 1.198 (IL-13) − 1.197 (IFN-γ).

In summary, using linear discriminant analysis, a model based on IL-13 and IFN-γ correctly predicted if an individual had been vaccinated or not, but failed to separate groups sampled at various time points after vaccination.

### Capacity of supernatants collected from *Ft*-stimulated cells to confer protection

**Figure 8:**
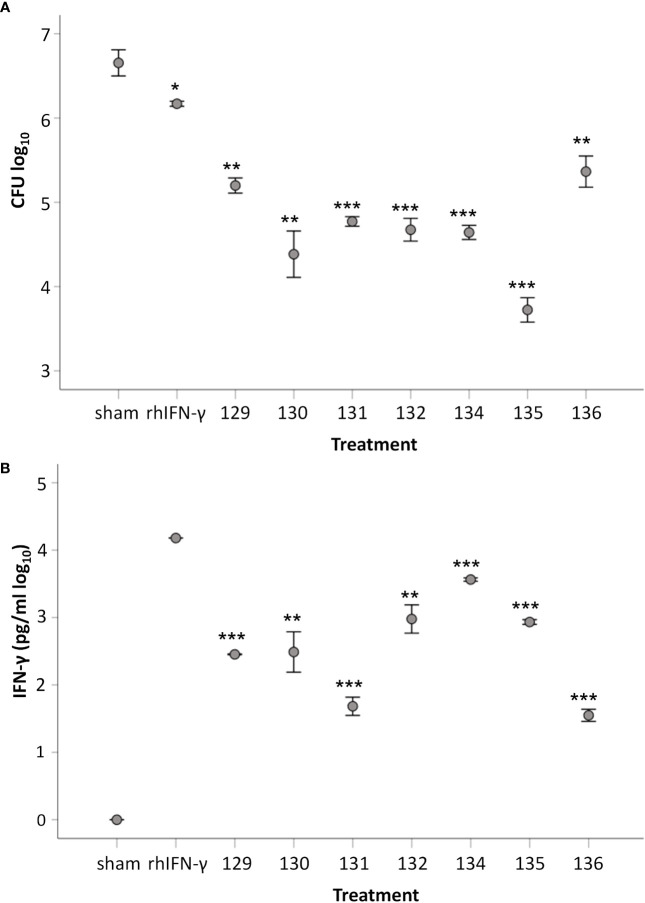
Capacity of supernatants from *Ft*-stimulated cells to induce protection against SCHU S4 in MDM. **(A)** Number of intracellular SCHU S4 after 24 h of infection. MDMs were infected with SCHU S4 and supernatants collected from *Ft*-stimulated cells from respective donor were added to separate cultures. Number of phagocytosed bacteria after 2 h of incubation with an MOI of 50 were 3.4 ± 0.02. **(B)** Concentration of IFN-γ in SCHU S4-infected cultures supplemented with supernatants collected from *Ft*-stimulated cells or with recombinant IFN-γ. The bars show the mean ± SEM of three observations. Statistical evaluation of differences between groups were analyzed using two-sided Student’s *t*-test. In diagram A, asterisksindicate significant differences vs. sham. In diagram B, asterisks indicate significant differences vs. cultures with recombinant IFN-γ (*p < 0.05, **p < 0.01, ***p < 0.001).

It was tested if the supernatants of stimulated cell cultures could activate monocyte-derived macrophages (MDMs) to control intracellular SCHU S4. Supernatants collected from *Ft*-stimulated cells from individual donors were added at a 20-fold dilution to cultures at the time of infection with SCHU S4. Concentration of the 17 cytokines in the 20-fold diluted supernatants varied among the individuals (**Table S3**). After 24 h, growth of SCHU S4 was reduced in all cultures supplemented with supernatants from *Ft* antigen-stimulated PBMCs compared to cultures with supernatants from sham-stimulated PBMCs (**Figure 8A**). The weakest growth inhibition of SCHU S4, 5-fold, was observed in cultures supplemented with supernatants from donor D129 (*p* < 0.05), and the strongest inhibition, 230-fold, was observed in cultures supplemented with supernatants from donor D135 (*p* < 0.001) (**Figure 8A**). Supernatants from all donors elicited a significantly better control of SCHU S4 in MDMs compared to recombinant IFN-γ (*p* < 0.05 for 136, *p* < 0.01 for 129, and *p* < 0.001 for all other donors, **Figure 8A**). The concentration of recombinant IFN-γ, 15 ng/ml, was at least 5-fold higher than the concentration of IFN-γ in any of the 20-fold diluted supernatants (**Figure 8B**). This suggested that the anti-*F. tularensis* effect elicited by IFN-γ was potentiated by other cytokines present in the supernatant. Potential candidates were IL-2, IL-7, MIP-1B, and TNF, the levels of which, as of IFN-γ, displayed a negative correlation to the number of bacteria in the cultures, i.e., higher concentrations correlated to lower bacterial numbers (*p* < 0.05, **Table 3**). Levels of IL-13 and G-CSF showed a positive correlation to the number of bacteria in the cultures (*p* < 0.05, **Table 3**).

**Table 3:** Correlation of intracellular bacteria to concentration of cytokines in supernatants.

	IL-2	IL-7	IL-13	G-CSF	IFN-γ	MIP-1β	TNF
CFU^1^	−0.635^2^*^3^	−0.576*	0.638*	0.599*	−0.643*	−0.853***	−0.782*

^1^ Colony-forming units, numbers of intracellular bacteria at 24 h.

^2^ Spearman´s Rho and Spearman’s rank correlation test were used to correlate numbers of intracellular bacteria after 24 h to cytokine concentrations in the supernatant. Cytokines not displaying significant correlation are not included in the table.

^3^ Asterisks indicate significant correlation between the indicated cytokine and numbers of intracellular bacteria. *p < 0.05, **p < 0.01, and ***p < 0.001.

Collectively, cells from vaccinated donors produced a mixture of cytokines in response to stimulation with *Ft* antigen that activated MDMs to control intracellular SCHU S4. The control elicited was significantly better than that conferred by recombinant IFN-γ.

## Discussion

T-cell-mediated memory immunity is dependent on a pool of memory cells. The half-life of vaccine-induced immunity is one or several decades; however, most evidence indicates that individual memory cells may be comparatively short-lived, in the order of months, and therefore the only logical explanation for the persistence of cell-mediated immunity for decades is the sustainability of clonal populations of memory cells (23). This is achieved by balancing proliferation, death, and differentiation rates of the populations. Thus, the cells within the populations confer long-lived memory rather than being long-lived memory cells. The longevity of these clonal populations is generally ill-defined and possibly distinct for a given T-cell population. An improved understanding of how long-term memory immunity is sustained is critical to improve efficacy of vaccines that rely on cell-mediated immunity.

Previously, we demonstrated that T-cell-mediated immune responses to *F. tularensis* may persist many decades after natural infection or vaccination (24). Herein, we elaborate on this finding and describe the cell-mediated immune responses quantitatively and qualitatively during a 1-year period after vaccination with the live vaccine strain of *F. tularensis*. Importantly, the experimental system used is devoid of antibodies; thus, any influence of humoral immunity can be excluded. Proliferative responses, cytokine secretion, and the intracellular cytokine profiles of CD4 and CD8 cells and memory cell populations were mostly indistinguishable when PBMCs were analyzed at the three early time points after vaccination. However, absolute levels somewhat decreased thereafter, although still significantly increased compared to the responses of the PBMC obtained before vaccination. Antigenic cross-reactivity is unlikely to sustain the longevity since *F. tularensis* is not closely related to other human pathogens (25). Moreover, since tularemia is a rare disease in most regions of the world (26), reexposure is an unlikely reason for the extremely long-lived cell-mediated immunity and, therefore, the data support the notion that natural infection, or vaccination, may result in very long-lasting, persisting for many decades. The narrow immunoreactivity to *F. tularensis* is distinct from that to many other infections due to its antigenic uniqueness and rare occurrence of tularemia. In the case of *F. tularensis*, there is no direct evidence that the long-lasting cell-mediated immunity confers protection against subsequent challenge; however, indirect evidence strongly indicates that this may be the case, since tularemia has been very rarely recorded in vaccinated individuals and only a handful of cases of reinfection have been reported during the last century (27, 28).

Beyond the characterization of the longevity of the cell-mediated immune responses, we also identified the cytokine profiles indicative of the vaccine-induced immune responses by intracellular staining of the PBMC T-cell populations. A more detailed analysis of subpopulations revealed that CD4 cell expression of intracellular IFN-γ was increased after vaccination and sustained for the whole period of 1 year. CD8 cells expressed increased levels of IFN-γ, IL-2, and MIP-1β after vaccination. All three cytokines have been identified in our previous studies of human vaccine-mediated immune responses (12, 22). When memory T-cell subpopulations were analyzed, the most nuanced cytokine expression was detected in the CD4 T_EM_ subpopulation with regard to MIP-1β and in the CD4 T_TM_ subpopulation with regard to IFN-γ, IL-2, and MIP-1β, since the expression of each of the cytokines was increased at all time points after vaccination. Also, the CD8 T_EM_ and T_TM_ subpopulations showed increased expression of IFN-γ, IL-2, and MIP-1β at all time points. Thus, the findings corroborate previously published data regarding the relevance of these cytokines in the memory immune response against *F. tularensis* after vaccination and demonstrate that the T_EM_ and T_TM_ populations exhibit the most diversified cytokine expression.

The identification of the CD4 T_TM_ and CD8 T_EM_ and T_TM_ subpopulations as the predominant reservoirs for cytokine secretion was not entirely surprising. The T_EM_ subpopulation is known to rapidly upregulate effector functions and to also express homing receptors for migration to nonlymphoid sites of inflammation and to possess high levels of gut-homing molecules and chemokine receptors (29). T_TM_ cells display an intermediate phenotype between T_EM_ and T_CM_ subpopulations, since some transcript expression levels closely align with those of T_EM_ cells, e.g., *CD62L* and *PIM2*, whereas others with those of T_CM_ cells, e.g., *FasL* and *IFN-γ* (30). Thus, the T_EM_ and T_TM_ subpopulations represent potential effector populations and the identification of their potent upregulation of multiple cytokines is therefore logical and identify them as important for the effective protective responses present after tularemia vaccination. In contrast, we did not detect the same broadly increased cytokine secretion by the T_CM_ and T_EMRA_ subpopulations. This reinforces the general concept that the T_CM_ subpopulation constitutes quiescent cells that require very strong stimulation and costimulation to respond to the cognate antigens (31). Their most important role with regard to protection against tularemia may be to serve as a reservoir that can be clonally expanded and differentiated into the T_EM_ and T_TM_ subpopulations. In fact, it cannot be ruled out that the *in vitro* antigen stimulation utilized in the present study led to differentiation of some T_CM_ cells to T_EM_ or T_TM_ cells. As for the T_CM_ subpopulation, the T_SCM_ subpopulation may also serve as a reservoir of cells for subsequent differentiation to effector memory cells. This would be in line with previous observations regarding the T_SCM_ subpopulation (32). The relevance of the T_EMRA_ subpopulation appears to be infection-dependent; e.g., HIV-specific T cells predominantly belong to the T_EM_ subpopulation, while CMV-specific T cells are mainly of the T_EMRA_ phenotype (33). Our findings indicate a rather modest role for the T_EMRA_ subpopulation after tularemia vaccination.

Linear discriminant analysis of patterns of secreted cytokines was utilized and a model was created based on the levels of IFN-γ and IL-13. This analysis revealed that discrimination between non-vaccination and vaccination was 100%, but that resolution between the groups after vaccination was low. This indicates that the qualitative and quantitative responses observed after vaccination did not change during the observation period of 1 year. This finding agrees with our previous studies demonstrating that recall responses are sustained for three decades without evidence of decline (22). Various types of logistical modeling, similar to the linear discriminant analysis used herein, have been used in previous studies on tularemia secreted cytokines, cytokine gene expression, and lymphocyte stimulation indices, and these have been linked to protective capacity or immune status (12, 13, 22, 24, 34). Also in the mouse model, similar modeling has been utilized and combined results from *in vivo* gene expression and a co-culture method (35). Such results from animal and human models of tularemia are highly relevant with regard to the FDA Animal Rule (36). The rule stipulates that vaccine efficacy and approval can be assessed based on data from animal models only, provided that protective mechanisms are well characterized and the animal data therefore can be extrapolated to the human situation. Thus, the aforementioned data fulfill the criteria and will form an important basis for the approval of new tularemia vaccines. The identification of IL-13 in the context is intriguing since it was long ago demonstrated that the cytokine inhibits nitric oxide production by activated macrophages and thereby most likely will also mitigate the protective responses to *F. tularensis* (37).

Our findings identified IFN-γ as a key cytokine being expressed at high levels after vaccination by both CD4 and CD8 T cells. This finding was not unexpected, since the crucial role of the cytokine for protection against tularemia was first identified more than 30 years ago using various animal models (38–45). Moreover, we have previously used a human co-culture model and demonstrated the protective ability of IFN-γ (12). In animal models, mechanisms dependent on guanylate-binding proteins GBP2 and GBP5 are crucial to effectuate the IFN-γ-mediated control (46, 47). This was first demonstrated for the closely related bacterium *F. novicida* and subsequently also for the LVS strain using infection of mononuclear cells; however, control of the highly virulent SCHU S4 strain was not observed in the model (48). Later, it was demonstrated that control of infection with each of the three *F. tularensis* strains occurred in a mouse co-culture model, but, again, control was critically dependent on GBPs (49). Thus, the evidence from animal models indicate that the control of highly virulent strains is distinct from that of attenuated *F. tularensis* strains and demonstrates that the use of such strains in the models is necessary to identify relevant correlates of immunity and protection.

The strategy used in the present study, to directly assess the protective ability of supernatants elicited during *F. tularensis*-specific immune responses allowed identification of cytokines that correlated to protection in the model. The supernatants demonstrated protective capability even at a 20-fold dilution. However, there were distinct individual differences and the inhibitory effects varied from 5-fold to 230-fold, but this was still as efficacious, or superior to the effect mediated by high levels of recombinant IFN-γ, despite the fact that this concentration of IFN-γ was at least 5-fold higher than the concentrations in the supernatants. Thus, the supernatants provided additional, strongly protective effects besides that of IFN-γ. Of relevance, the levels of several other cytokines were found to correlate to the degree of protection observed. Specifically, IL-2, MIP-1β, TNF, and IL-7 all fulfilled the criterion and the former three of these cytokines have previously been observed to correlate the control of infection in a human co-culture model and also in an animal models of tularemia (12, 34, 50, 51). The strategy was found to be useful and can later be combined with direct assessment of the contribution of individual cytokines, e.g., by depletion of one or several together with the assessment of the protective ability or by supplementation of combinations of recombinant cytokines.

The findings herein serve to identify both correlates of immunity and protection. In fact, even correlates of immunity are very challenging to identify with regard to cell-mediated immune responses. In the case of the most common global infection, tuberculosis, the identification of potential immune correlates is challenging, e.g., the relevance of multifunctional cytokine-producing T cells as correlates has been questioned (52), although there are still considerable efforts trying to identify such correlates (53). With regard to tularemia, most work to identify correlates has been based on animal models, particularly the mouse model. A general finding has been the identification of Th1-related cytokines in these models; e.g., the demonstration of increased levels of IFN-γ, TNF, and MCP-1 has been consistent. Moreover, the levels of these cytokines correlated to protection after vaccination with attenuated *F. tularensis* mutants (34, 35). Studies on immunity after tularemia vaccination, or natural infection of humans, have identified multifunctional T cells similar to findings described for tuberculosis patients (22, 52, 54). When a human co-culture system was used, correlations between levels of IFN-γ, TNF, and MIP-1β and protection were observed (12). Thus, there is substantial indirect evidence that levels of Th1 cytokines, such as IFN-γ, TNF, and MIP-1β, are correlates of protection in various animal and human tularemia models, thereby in much agreement with the present findings (12, 22, 34, 35, 54). Of note, none of the studies on *F. tularensis* has included neutralization of cytokines as a direct demonstration of their contribution to protection.

The present study exclusively utilized PBMCs, and this may affect the results, since memory immunity expressed by this cell type is sometimes distinct compared to that of tissue-resident immune cells. For example, it has been demonstrated that mouse lung T cells require repeated antigen stimulation to sustain memory immunity for 1 year, whereas memory immunity among circulating T cells was preserved during the same time without restimulation (55). Also, with regard to tularemia, evidence indicates that systemic and organ-specific cell-mediated immunity is distinct, e.g., an attenuated mutant of *F. tularensis* conferred superior protection after intranasal vs. intradermal vaccination (35). It was hypothesized that there are organ-specific differences between immune cells and therefore certain routes of immunization confer optimal protection. If memory immunity to some extent will be cell-specific, this may be a caveat when characterizing human protective immunity since cell sources other than peripheral blood will be severely limited. It should be noted that although intranasal vaccination can confer immunological benefits, there may be an increased risk of adverse events with this route as shown for certain vaccines (56).

Collectively, the findings herein identify characteristics of long-term immunospecific T cells, including T_EM_ and T_TM_ subpopulations secreting an array of cytokines, following vaccination against *F. tularensis*. Moreover, individual cytokines were identified, the levels of which correlated with the degree of protection. Thus, the data provide important information about memory T cells and effector mechanisms that form the basis for the protective mechanisms operative against *F. tularensis.*

## Data availability statement

The original contributions presented in the study are included in the article/**Supplementary Material**. Further inquiries can be directed to the corresponding author.

## Ethics statement

The studies involving humans were approved by Swedish Ethical Review Authority, permits 2019-01567 and 2020-01860. The studies were conducted in accordance with the local legislation and institutional requirements. The participants provided their written informed consent to participate in this study.

## Author contributions

HL, KE, and IG performed the experiments. AS and HL designed the study. HL, KE, and AS analyzed the data and wrote the manuscript. HL, KE, IG, CG, and AS reviewed the manuscript. All authors contributed to the article and approved the submitted version.
