# Supplementary material for: Analyses of human immune responses to Francisella tularensis identify correlates of protection
Source: Front Immunol. 2023 Sep 15;14:1238391. doi: 10.3389/fimmu.2023.1238391 (PMC10540638; doi:10.3389/fimmu.2023.1238391)
Supplement: Supplementary file 1 [file DataSheet_1.pdf]

Supplementary information

Table S1. Analysis of differences among groups of multifunctional cells[1]

Cytokines	CD8 T_{EM}	CD8 T_{TM}
IFN-γ+IL-2+MIP-1β+TNF	0.486[2]	0.725
IFN-γ+IL-2+MIP-1β	0.001	0.001
IFN-γ+IL-2+TNF	0.509	0.723
IFN-γ+IL-2	0.003	0.003
IFN-γ+MIP-1β+TNF	0.004	0.002
IFN-γ+MIP-1β	0.000	0.000
IFN-γ+TNF	0.010	0.293
IFN-γ	0.000	0.000
IL-2+MIP-1β+TNF	0.410	0.332
IL-2+MIP-β	0.036	0.038
IL-2+TNF	0.615	0.601
IL-2	0.000	0.000
MIP-1β+TNF	0.066	0.014
MIP-1β	0.027	0.095
TNF	0.415	0.382

[1] One-Way ANOVA was used to test if there were differences among the groups, pre-vaccination, two, four, 12 and 52 weeks postvaccination, with regard to number of cells co-expressing various combinations of cytokines.

[2]P-value retrieved from the One-Way ANOVA.

Table S2. Analysis of differences among groups of multifunctional cells[1]

Multifunctionality	week	test week	CD8 T_{EM}	CD8 T_{TM}
IFN-γ/IL-2/MIP-1β	0	2	0.040[2]	0.136
		4	0.002	0.001
		12	0.010	0.002
		52	1.000	0.343
	2	4	1.000	1.000
		12	1.000	1.000
		52	1.000	1.000
	4	12	1.000	1.000
		52	0.244	1.000
	12	52	0.676	1.000
IFN-γ/MIP-1β	0	2	0.016	0.016
		4	0.000	0.000
		12	0.004	0.000
		52	0.682	0.089
	2	4	1.000	1.000
		12	1.000	1.000
		52	1.000	1.000
	4	12	1.000	1.000
		52	0.264	1.000
	12	52	1.000	1.000

[1] One-Way ANOVA with Bonferroni Post hoc test was used to test if there were differences among the groups, pre-vaccination, two, four, 12 and 52 weeks postvaccination, with regard to number of cells co-expressing various combinations of cytokines.

[2]P-value retrieved from the One-Way ANOVA.

Table S3. Cytokine levels in 20-fold diluted supernatants, collected from recall-stimulated PBMC

Cytokine	Donor						
	129	130	131	132	134	135	136
IL-1B	2.20	1.58	1.40	1.62	2.29	2.16	1.50
IL-2	0.95	1.04	0.57	0.97	1.18	1.16	0.56
IL-4	0.57	0.42	0.26	0.54	0.75	0.62	0.28
IL-5	1.58	1.44	1.42	1.53	1.65	1.57	1.44
IL-6	3.19	3.21	3.30	3.39	3.29	3.39	3.34
IL-7	0.28	0.21	0.07	0.52	0.74	0.55	-0.59
IL-8	5.68	6.12	6.86	6.09	5.60	6.86	6.86
IL-10	0.34	0.15	0.35	0.33	0.42	0.37	0.21
IL-12p70	0.74	0.49	-0.12	0.55	0.97	0.66	0.15
IL-13	-0.25	-0.34	0.32	-0.01	-0.01	-0.62	0.42
IL-17	1.20	0.88	0.79	0.92	1.31	1.19	0.75
GCSF	1.49	1.73	1.97	1.68	1.55	1.40	1.92
GMCSF	0.68	0.99	1.36	0.64	0.87	0.53	1.34
IFN-γ	2.45	2.49	1.68	2.98	3.56	2.93	1.55
MCP-1	2.99	3.07	3.19	3.09	3.12	3.61	3.24
MIP-1B	2.63	2.83	2.66	3.11	2.76	2.93	2.63
TNF	2.69	2.97	2.35	2.85	3.03	3.07	2.28

[1]Cytokine content (pg/ml) was determined by Multiplex Cytokine Analysis in supernatants obtained from *Ft*-stimulated cultures with PBMC collected 52 weeks after vaccination.

Figure S1

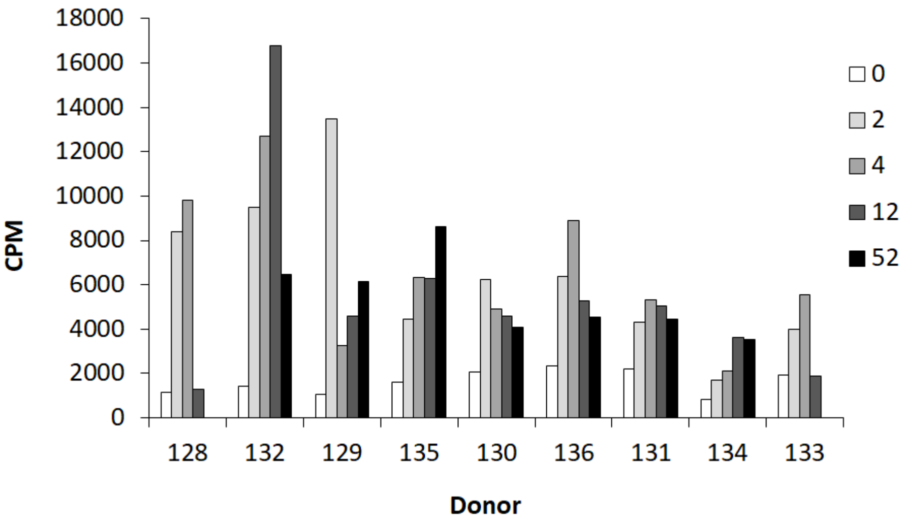

Fig. S1. Proliferative responses of PBMC after recall stimulation with *Ft*-antigen. PBMC collected from individuals, before vaccination (0) and two, four, 12, and 52 weeks after vaccination, were cultured in the presence of *Ft*-antigen for three days. Six h after addition of tritium-thymidine, incorporation was measured as CPM. The mean of triplicate samples is shown for each individual and time point.

Figure S2

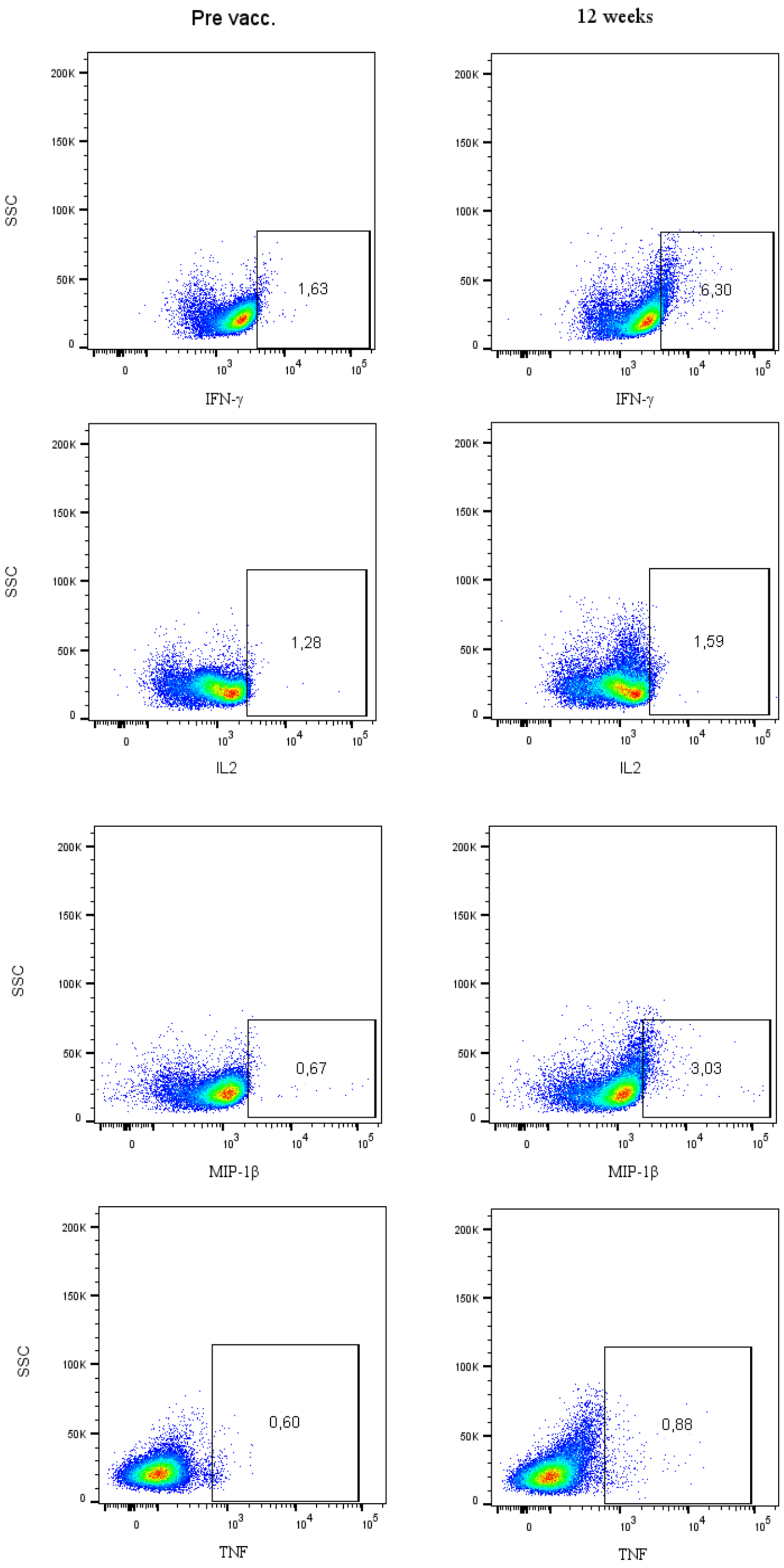

Figure S2. Representative gating of intracellular cytokines in the CD8 population.

Figure S3

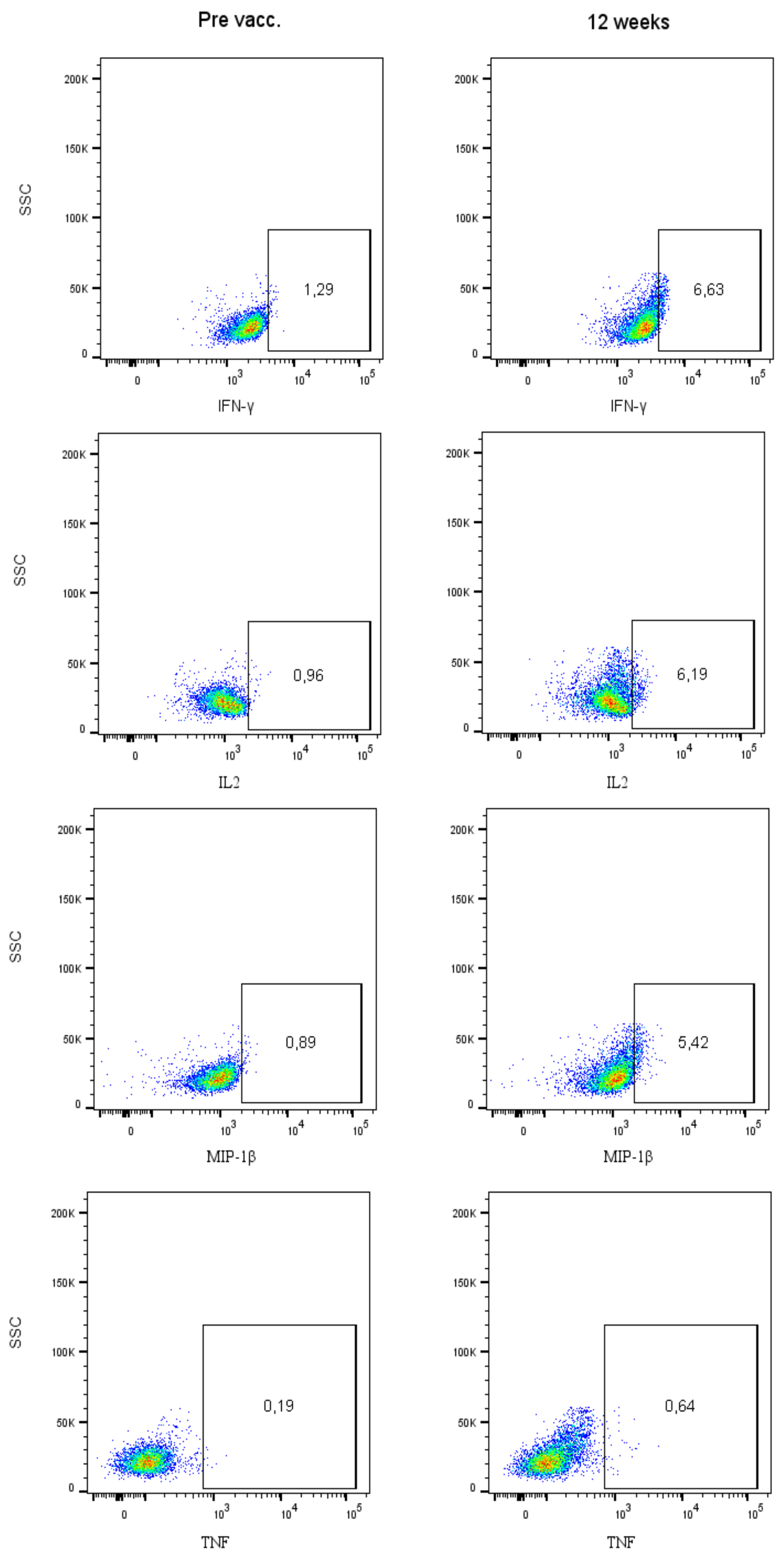

Figure S3. Representative gating of intracellular cytokines in the CD8 T_{TM} population.

Figure S4

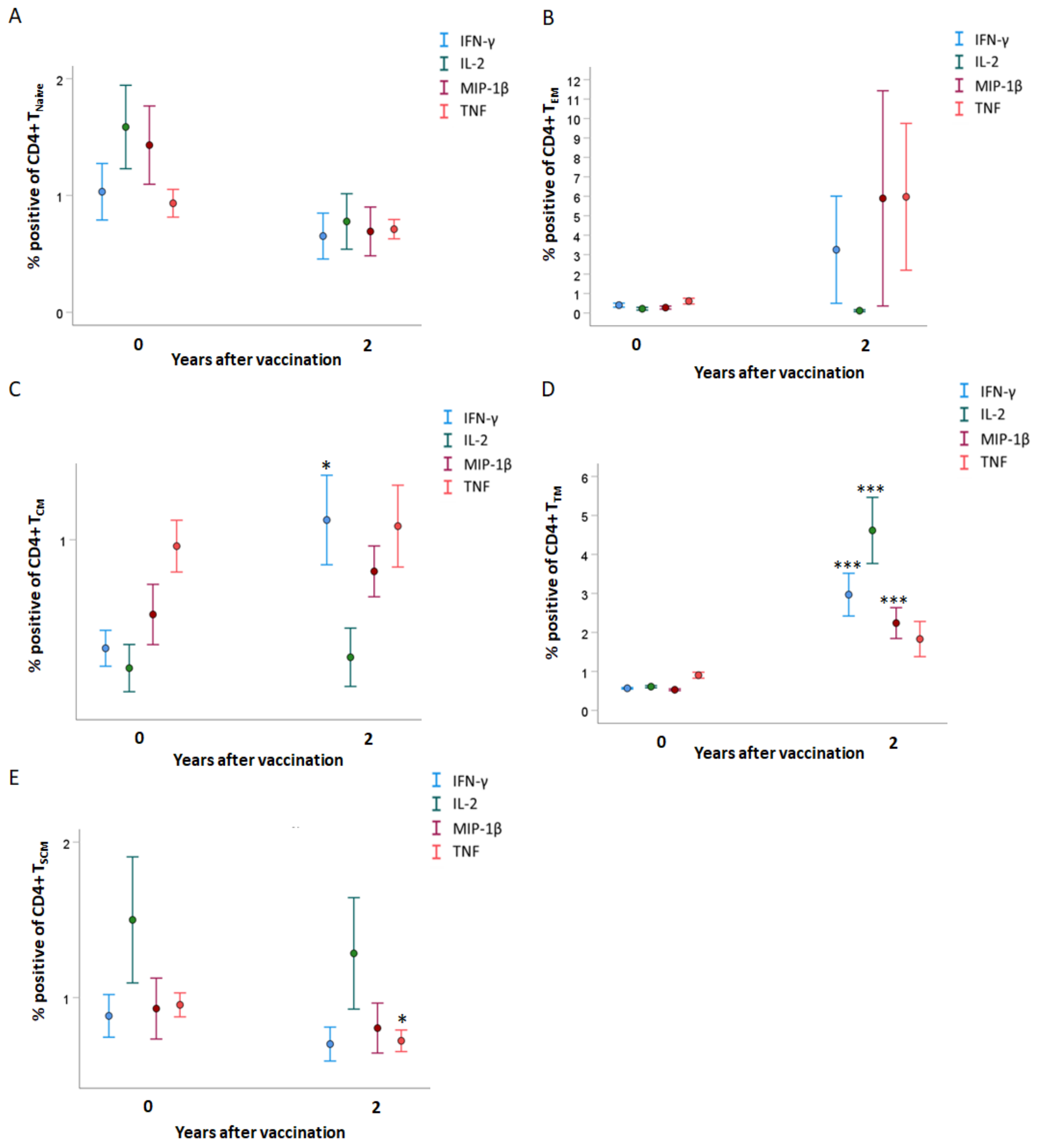

Fig. S4. FACS analysis of intracellular cytokine expression of CD4 memory subpopulations. PBMC collected from seven individuals before vaccination (0) and two years (2) after vaccination were recall stimulated with *Ft* antigen for three days. The cells were stained to detect the following CD4 memory populations A) T_{Naive}, B) T_{EM}, C) T_{Cm}, D) T_{TM} and E) T_{SCM} and their expression of IFN-γ, IL-2, MIP-1β, or TNF. The mean ± SEM of triplicate samples from seven individuals are shown for each time point and cytokine. Stars indicate significant differences of intracellular cytokine expression compared to time point 0. The T_{EMRA} subpopulation was not detected in the CD4+ population.

Figure S5

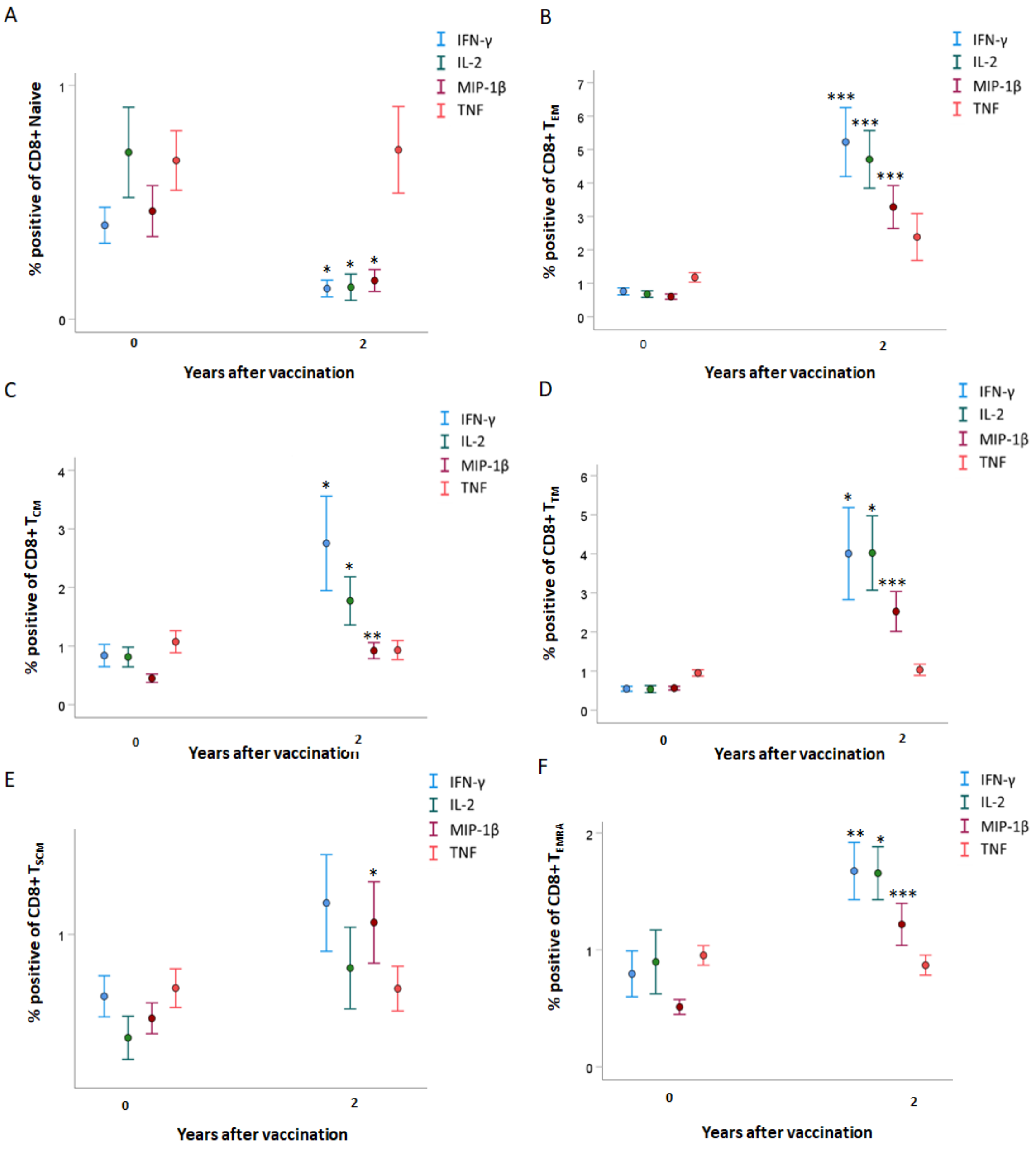

Fig. S5. FACS analysis of intracellular cytokine expression of CD8 memory subpopulations. PBMC collected from seven individuals before vaccination (0) and two years (2) after vaccination were recall stimulated with *Ft* antigen for three days. The cells were stained to detect the following CD4 memory populations A) T_{Naive}, B) T_{EM}, C) T_{Cm}, D) T_{TM}, E) T_{SCM} and F) T_{EMRA} and their expression of IFN-γ, IL-2, MIP-1β, or TNF. The mean ± SEM of triplicate samples from seven individuals are shown for each time point and cytokine. Stars indicate significant differences of intracellular cytokine expression compared to time point 0.

Figure S6

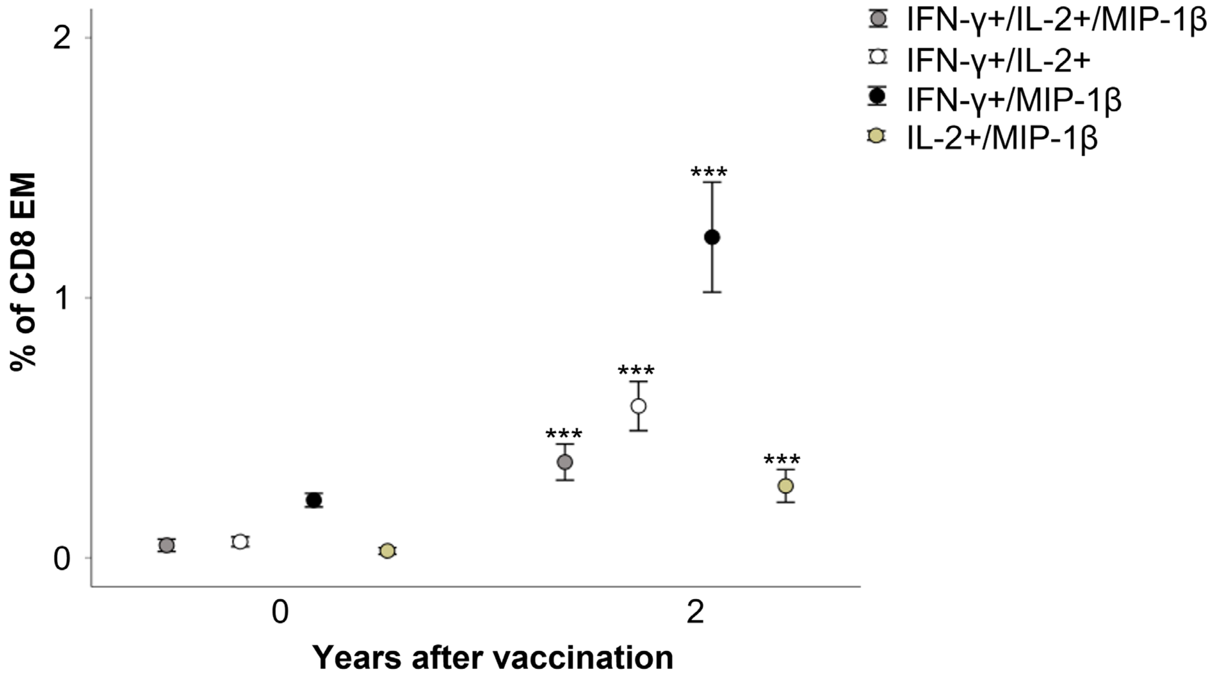

Fig. S6. Multifunctional CD8 T_{EM} memory cell populations. Data was subjected to Boolean gating in order to detect multifunctional memory cell populations. The mean ± SEM of triplicate samples from seven individuals are shown for each time point and cytokine combination. Stars indicate significant differences of number of cells positive for intracellular cytokine expression compared to time point 0.
